# Opto-thermoelectric pulling of light-absorbing particles

**DOI:** 10.1038/s41377-020-0271-6

**Published:** 2020-03-06

**Authors:** Linhan Lin, Pavana Siddhartha Kollipara, Abhay Kotnala, Taizhi Jiang, Yaoran Liu, Xiaolei Peng, Brian A. Korgel, Yuebing Zheng

**Affiliations:** 1 Walker Department of Mechanical Engineering, The University of Texas at Austin, Austin, TX 78712 USA; 2 Materials Science & Engineering Program and Texas Materials Institute, The University of Texas at Austin, Austin, TX 78712 USA; 3 State Key Laboratory of Precision Measurement Technology and Instruments, Department of Precision Instrument, Tsinghua University, Beijing, 100084 People’s Republic of China; 4 McKetta Department of Chemical Engineering, The University of Texas at Austin, Austin, TX 78712 USA; 5 Department of Electrical and Computer Engineering, The University of Texas at Austin, Austin, TX 78712 USA

**Keywords:** Optical manipulation and tweezers, Optofluidics

## Abstract

Optomechanics arises from the photon momentum and its exchange with low-dimensional objects. It is well known that optical radiation exerts pressure on objects, pushing them along the light path. However, optical pulling of an object against the light path is still a counter-intuitive phenomenon. Herein, we present a general concept of optical pulling—opto-thermoelectric pulling (OTEP)—where the optical heating of a light-absorbing particle using a simple plane wave can pull the particle itself against the light path. This irradiation orientation-directed pulling force imparts self-restoring behaviour to the particles, and three-dimensional (3D) trapping of single particles is achieved at an extremely low optical intensity of 10^−2^ mW μm^−2^. Moreover, the OTEP force can overcome the short trapping range of conventional optical tweezers and optically drive the particle flow up to a macroscopic distance. The concept of self-induced opto-thermomechanical coupling is paving the way towards freeform optofluidic technology and lab-on-a-chip devices.

## Optical pulling: Light can move particles against the stream

In a phenomenon termed opto-thermoelectric pulling (OTEP), the optical heating of a particle due to the absorption of a simple plane light wave can pull the particle against the direction of the light path. Linhan Lin, Yuebing Zheng and colleagues at the University of Texas at Austin, USA, demonstrate this counter-intuitive concept by manipulating particles of silicon 500 namometers across. The authors propose a mechanism allowing the effect of optical heating to oppose and overcome the optical force in the direction of the light beam. They explain how this can be achieved by manipulating the interaction of the energy of photons with the target particles. The phenomenon can trap and move particles using extremely low optical intensities. There are potential applications based on the interaction of light with fluids (optofluidics) especially in lab-on-a-chip devices.

## Introduction

A photon carries momentum, which can be transferred to low-dimensional objects to realize optical manipulation. Laser radiation can push a particle along the light path once the particle ‘feels’ the radiation pressure. Engineering of a laser beam using a high numerical aperture (NA) lens creates an intensity gradient that can efficiently trap an object at the beam centre, which was developed as optical tweezers by Arthur Ashkin^1^. However, the idea that light can pull an object against the flow of light, which is also known as an optical tractor beam, is counter-intuitive because the Poynting vector normally points along the propagation direction of incident light^2^. To maintain the conservation of momentum, the key to achieving optical pulling is to engineer the sign of the momentum change $\Delta \mathop{{\boldsymbol{p}}}\limits^{\rightharpoonup}$ during the light–matter interaction. Over the past decade, some strategies have been proposed to achieve optical pulling, such as sign reversal of the Poynting vector^3,4^, amplification of the forward-to-backward scattering intensity to transfer backward momentum to the objects^5–8^, or the interaction between the object and the self-collimation mode from the photonic crystals^9^. However, it can be proven that optical pulling based on momentum transfer between an incident plane wave and low-dimensional objects is unachievable.

From another perspective, photons also carry energy, which can be transferred to low-dimensional objects. Specifically, photon-to-phonon conversion, also known as the optothermal effect, is an entropically favourable process. Laser radiation on a light-absorbing object creates a temperature difference with asymmetric thermal energy densities, which provides an alternative strategy for optical manipulation^10^. Photophoresis, which arises from the asymmetric gas-dynamic force when a light-absorbing object is directionally irradiated in the gaseous medium, drives the object towards the low-thermal-energy side, where the force imparted by the gas molecules is weaker^11^. It is comprehensible that the photon-to-phonon energy transfer predominates at the illuminated side of light-absorbing objects. The key to pulling an object using photophoresis is to flip the thermal energy distribution, which requires a rigorous design for both the heating optics and the structures of the light-absorbing objects^11–13^. To date, pulling an object in free space using simple optics is still elusive, which hinders the development of the optical pulling force as a general manipulation technology.

The essential approach to overcoming the rigorous design rule in the existing optical pulling systems is to obtain a light-directed force pointing from the side with low thermal energy density to the side with high thermal energy density. Herein, we propose that the directional irradiation of an incident plane wave on a light-absorbing particle can create a temperature gradient on the particle surface, which drives the thermophoresis of ionic species and induces a thermoelectric field to pull the particle consistently. Specifically, we demonstrate that the opto-thermoelectric pulling (OTEP) force can impart self-restoring behaviour to the particle for low-power three-dimensional (3D) manipulation. Moreover, we prove that OTEP can overcome the short working range of conventional optical tweezers and optically drive the particle flow up to a macroscopic distance without the need for mechanical confinement or pumping.

## Results

### Working principle

**Fig. 1:**
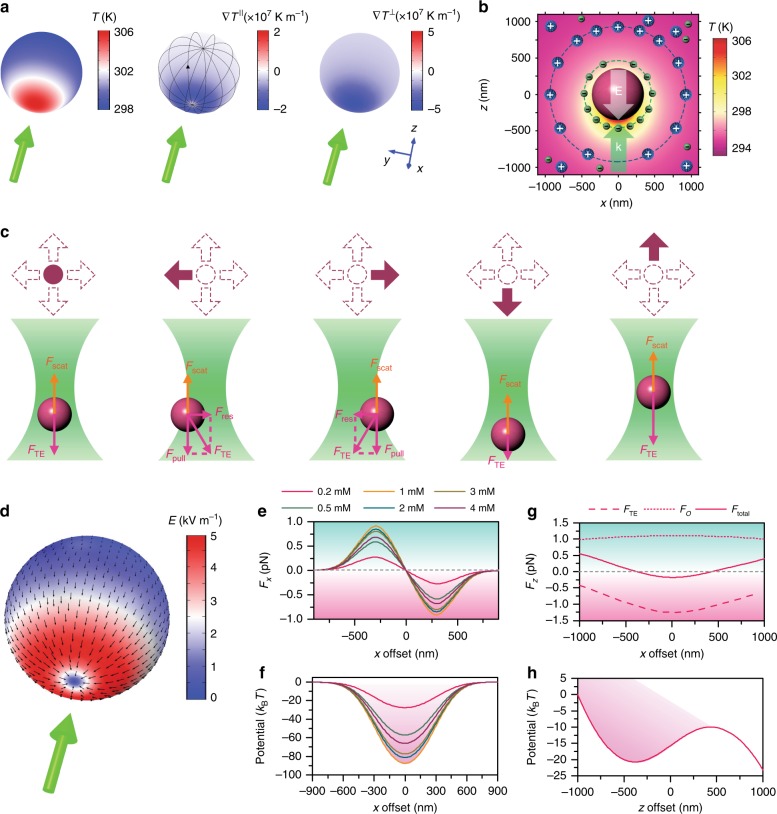
Working principle of OTEP. **a** Temperature (left), parallel temperature gradient (middle) and perpendicular temperature gradient (right) on the surface of a 700 nm Si particle irradiated by an incident laser beam (indicated by a green arrow). **b** Schematic showing the ionic distribution under the temperature field and the resultant thermoelectric field on the Si particle irradiated by an incident laser beam. The green arrow indicates the wave vector **k** of the incident laser beam, while the white arrow gives the direction of the thermoelectric field **E**. **c** Force analysis on the Si particle at different locations in relation to the focused heating laser beam. On the top panel, the solid circle (leftmost) indicates that the Si particle is at the trapping centre, which is on the beam axis and below the focal plane, while the solid arrows (all others) indicate the offset directions of the Si particle relative to the trapping centre. It should be noted that the laser beam was launched from the bottom. **d** The calculated thermoelectric field distribution on the Si particle surface at a CTAC concentration of 1 mM. **e** The in-plane (i.e., *xy* plane in **a**) trapping force *F*_*x*_ as a function of the in-plane offset (i.e., *x* direction in **a**) at different CTAC concentrations. **f** The in-plane trapping potentials at different CTAC concentrations. **g** The out-of-plane (i.e., *xz* plane in **a**) force *F*_*z*_ as a function of the out-of-plane offset (i.e., *z* direction in **a**) at a CTAC concentration of 1 mM. The dashed curve represents the thermoelectric force, the dotted curve represents the optical force, and the solid curve represents the total force. **h** The out-of-plane trapping potential at a CTAC concentration of 1 mM. The filled colour means that the potential is asymmetric (i.e., deeper to the left or below the focal plane). The heating laser has a wavelength of 532 nm, an optical power of 60 μW and a beam diameter of 2 μm at the focal plane

Generally, thermophoresis describes the directional migration of an object along an external temperature gradient field, which can be generated optically or electrically^14,15^. It provides another possibility to convert photon energy into the mechanical energy of low-dimensional objects^16–21^. It is also possible to manipulate a low-dimensional object using the temperature gradient generated by the object itself without external heating sources, which is termed self-thermophoresis^22^. Herein, we adopt amorphous Si particles (see Fig. S1 for a scanning electron microscopic images), which possess high optothermal conversion efficiency and low thermal conductivity. Upon laser irradiation, a temperature gradient field is built on the Si particle surface due to inhomogeneous heating. Unlike Janus particles, in which the orientation of the temperature gradient is geometrically determined, the orientation of the temperature gradient on the Si particle surface depends only on the incident direction of the heating laser beam^23^. The left panel in Fig. 1a shows the overall surface temperature of a 700 nm Si particle irradiated by a 532 nm laser beam at an optical power of 60 μW in a water environment. We can see that a significant temperature difference of ~8 K is obtained between the hot and cold poles due to the low thermal conductivity of amorphous Si (1.8 W/m K). The corresponding surface temperature gradient $\nabla T^\parallel$ (middle panel) points from the rear pole to the illuminated pole. In contrast, the perpendicular component $\nabla T^ \bot$ always points towards the particle centre (right panel).

To generate the opto-thermoelectric field, we added an ionic surfactant (cetyltrimethylammonium chloride, CTAC) into the solution, where the CTAC micelles formed above the critical micelle concentration act as macro-cations, while the counter-Cl^−^ ions act as anions in the system^20,24^. Upon optical heating, the heat generated by the Si particle is transferred to the surrounding water molecules, with the temperature distribution illustrated in Fig. 1b. Both the macro-ions and counter-anions undergo thermophoretic migration from hot to cold. Since the CTAC micelles have a larger Soret coefficient (~10^−2^ K^−1^) than that of the Cl^−^ ions (7.18 × 10^−4^ K^−1^)^25–28^, the concentration gradient built along the temperature gradient is higher for the CTAC micelles. It can be schematically understood that the CTAC micelles stay in the cold region, while the Cl^−^ ions stay in the hot region, as displayed in Fig. 1b. The spatial separation between the macro-cations and the counter-anions gives rise to the thermoelectric field **E** pointing from the cold pole to the hot pole, contrary to the wave vector **k** of the incident laser. Considering the positive surface charge of the Si particle after CTAC adsorption, the Si particle is visually pulled towards the laser source by the self-generated thermoelectric field. In other words, the laser heating of the Si particle creates a thermoelectric field, which pulls the particle towards the light source.

It should be noted that the Si particle has a spherically symmetric shape and composition and that the orientation of the opto-thermoelectric field depends only on the optical heating site of the particle, i.e., the location of the particle in relation to the incident heating laser (see Fig. S2). We speculate that if the heating laser beam has a Gaussian intensity distribution, the particle can be trapped in 3D by its self-generated temperature gradient. Figure 1c shows the qualitative force analysis of a single Si particle when it is heated by a loosely focused laser beam. The out-of-plane (i.e., *xz* plane in Fig. 1a) pulling force *F*_pull_ is important to balance the optical scattering force *F*_scat_ in 3D trapping. An in-plane (i.e., *xy* plane in Fig. 1a) restoring force *F*_res_ will appear once there is an offset between the sphere centre and the beam axis. Specifically, an in-plane component of the thermoelectric force *F*_TE_ acts as the restoring force to pull the particle back to the beam centre. For a loosely focused laser beam, the out-of-plane scattering force can be treated as constant, and the optical gradient force is negligible. However, the temperature gradient is a function of the out-of-plane location of the Si particle. Briefly, the temperature gradient reaches the maximum when the particle centre overlaps with the focal plane and decays when the particle moves upwards or downwards. Thus, the out-of-plane balance site of the particle is located below the focal plane, where the OTEP force increases/decreases when the particle moves upwards/downwards. In other words, the net force of the OTEP force and the optical scattering force acts as the out-of-plane restoring force to keep the particles in the trap.

The opto-thermoelectric field can be calculated based on the ionic distributions surrounding the particle^29^. Figure 1d shows the thermoelectric field on the particle surface at a CTAC concentration of 1 mM, with a maximum electric field intensity above 5 kV m^−1^ (see Note S1 for the calculation detail). The surface charge densities of the Si particles are estimated from the zeta-potential measurements and applied to calculate the thermoelectric force. We further calculate the trapping force as a total of the thermoelectric force and the optical force. The in-plane trapping force as a function of the particle-to-beam-centre offset at different CTAC concentrations is illustrated in Fig. 1e. The in-plane trapping force varies at different in-plane locations, and it behaves as if the particle ‘prefers’ to be heated. When the CTAC concentration increases from 0.2 to 1 mM, the in-plane trapping force continuously increases due to the increase in both the thermoelectric field and the zeta potential of the Si particle. A maximum trapping force of 1 pN is obtained at 1 mM, which corresponds to a trapping depth of 87.4*k*_B_*T*. However, above 1 mM, the surface adsorption of CTAC molecules becomes saturated, and further addition of surfactant molecules will improve the ionic strength, collapsing the double layer and diminishing the zeta potential of the particles^30,31^. Hence, when the CTAC concentration increases from 1 to 4 mM, the trapping force successively decreases. It should be noted that the influence of the zeta potential on the trapping stiffness was also observed in the optical trapping of dielectric nanoparticles when the particle size was much smaller than the trapping wavelength and the particles could be approximated as point dipoles^32,33^. The out-of-plane force at a CTAC concentration of 1 mM is also analysed (Fig. 1g). We can see that the optical scattering force is not sensitive to the particle location along the *z*-axis, while a maximum out-of-plane pulling force is observed when the particle is at the focal plane (*z* = 0 nm). An equilibrium point is observed at *z* = −390 nm, where the total force on the particle is zero, and a restoring force is exerted on the particle once the particle deviates from the equilibrium point (Fig. 1g). It should be noted that the out-of-plane trapping potential is asymmetric (Fig. 1h), indicating that it is more stable to move the particle upwards (i.e., along the light path). From the out-of-plane trapping force and trapping potentials as functions of the CTAC concentration (Fig. S3), we can see that 3D opto-thermoelectric trapping is achievable in a 1 mM CTAC solution. In other words, it is possible to achieve self-opto-thermoelectric trapping (SOTET) of the Si particle by harnessing the heat generated on the particle.

### Self-opto-thermoelectric trapping

**Fig. 2:**
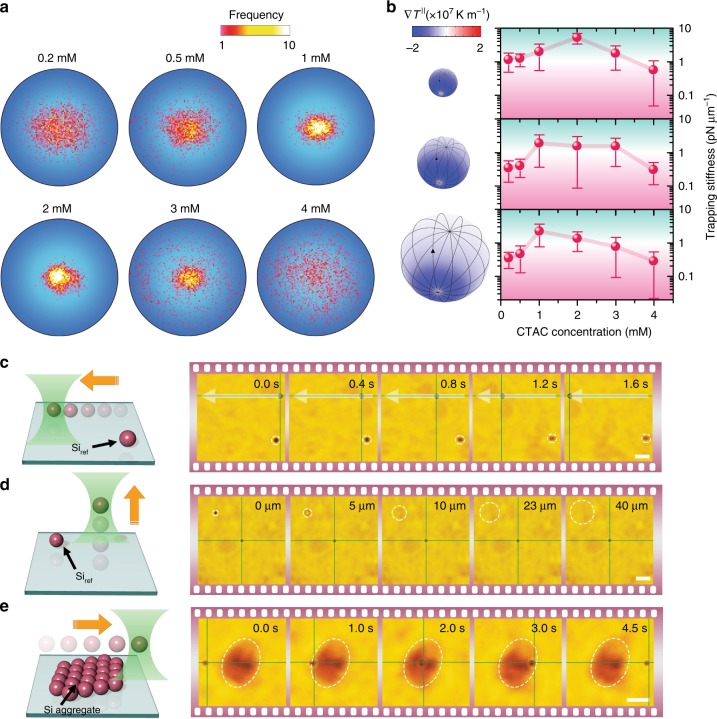
SOTET and 3D manipulation of single Si particles. **a** The position distribution histograms of a single 500 nm Si particle trapped by a single laser beam. **b** The parallel temperature gradient on single Si particles (left) and the corresponding trapping stiffness measured at different CTAC concentrations (right). Particle diameters: 500 nm (top); 700 nm (middle); and 1.2 μm (bottom). **c** A schematic (left) and optical images (right) show the in-plane dynamic delivery of a single 1.2 μm Si particle. **d** A schematic (left) and optical images (right) show the out-of-plane dynamic delivery of a single 500 nm Si particle. **e** A schematic (left) and optical images (right) show the trapping of a single 700 nm Si particle in 3D and the delivery of the particle across an aggregate of Si particles on the substrate. The dashed circles or ellipses show the individual particles or aggregate as visual references on the glass substrate. The grey arrows in **c** show the trajectory of the particle. The intersection of the green lines indicates the location of the laser beam. The trapping laser beam with a wavelength of 532 nm, an optical power of 60 μW, and a diameter of 2 μm at the focal plane was launched from the bottom. Scale bar: 5 μm (**c** and **e**); 10 μm (**d**)

Based on the proposed working principle of the SOTET effect, we carried out a proof-of-concept experimental study. As shown in Fig. 2a, we used a 532 nm laser beam with an optical intensity of 19 μW μm^−2^ to achieve SOTET of single Si particles on a glass substrate and tracked the particle locations during trapping. The representative position distribution histograms of a 500 nm Si particle as a function of the CTAC concentration are given in Fig. 2a (see also Video S1), with a narrow distribution observed at 1 and 2 mM and the broadest distributions observed at 0.2 and 4 mM. We further calculated the trapping stiffness of Si particles with different sizes based on the position distribution histograms (as shown in Fig. 2b), which is consistent with our theoretical analysis (Fig. S4). Interestingly, the smaller particles experience a higher trapping stiffness at the same optical intensity, which contradicts what occurs in traditional optical trapping. To investigate the underlying mechanism of the ‘abnormal’ correlation between the particle size and trapping stiffness, we simulated the surface temperature distribution of the Si particles for three different sizes. As shown in Fig. S5, the heating effect of the 500 nm Si particle is significant where the temperature continuously decreases from the illuminated pole to the rear pole. However, when the particle size increases, the heating effect is weakened due to the larger amorphous silicon volume, which disperses the heat more effectively. For the 1200 nm Si particle, most of the surface area has a constant temperature, i.e., the effective temperature gradient on the particle surface is significantly low (Fig. 2b). Thus, the thermoelectric field that pulls and traps the particle is localized around the illuminated pole, and the trapping force becomes limited for larger particles.

Based on the SOTET effect, we further demonstrate the dynamic manipulation of single Si particles through steering the laser beam. As shown in Fig. 2c, a single 1.2 μm Si particle is trapped on the glass substrate and transported horizontally at a velocity of ~23 μm s^−1^ (see also Video S2). More importantly, as discussed in Fig. 1g, h, 3D trapping is conceived by balancing the optical scattering force with the out-of-plane thermoelectric pulling force. To demonstrate the 3D manipulation capability, we irradiated a single laser beam onto a single 500 nm Si particle through an objective. By continuously moving the objective upwards, we can see that the illuminated particle moved upwards and stayed at the focal plane, while the reference particle fixed on the glass surface became out of focus (Fig. 2d; see also Videos S3 and S4 for the 3D manipulation of single Si particles). It should be noted that an oil objective lens with an NA of 0.5 was used for this demonstration. It is known that 3D manipulation can be achieved using traditional optical tweezers with a high-NA objective lens and a high optical power. However, the working distance of a high-NA objective lens is short, and thus, the out-of-plane operation range of the tweezers is limited. To better demonstrate the long operation distance in SOTET with simple optics, we used a ×40 air objective lens (NA = 0.75) to trap a single 500 nm Si particle and to transport the particle from the glass substrate to the top coverslip, covering a distance of ~126 μm (Fig. S6 and Video S5). It should be noted that the out-of-plane transport distance depends on the working distance of the objective lens and can be further extended when a thicker sample chamber is used. To further demonstrate the robustness of SOTET with simple optics, we trapped a single 700 nm Si particle in 3D and moved the particle across an aggregate of Si particles on the glass substrate, during which the trapped particle was retained even when the heating laser beam was partially blocked by the aggregate of Si particles (Fig. 2e and Video S6).

### Opto-thermoelectric shuttles

**Fig. 3:**
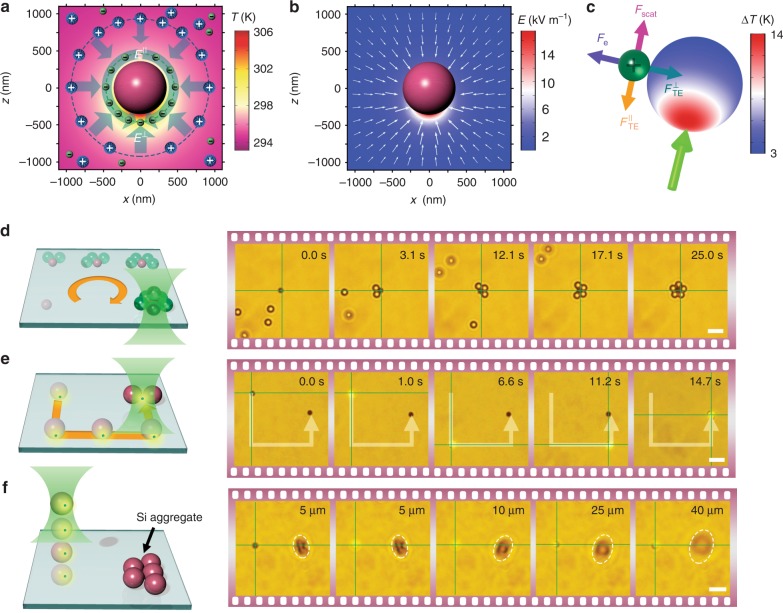
Opto-thermoelectric shuttle for low-power optical transport of non-absorbing particles with simple optics. **a** The simulated temperature distribution and schematic of the opto-thermoelectric field outside an optically irradiated Si particle. The blue arrows indicate the thermoelectric field pointed towards the Si particle centre for trapping, while the green arrows show that the thermoelectric field directs the motion of the trapped particles along the Si shuttle surface to the illuminated pole. **b** The simulated opto-thermoelectric field outside the Si particle. **c** Force analysis of a non-absorbing particle around an optically heated Si particle. **d** A schematic (left) and optical images (right) show the opto-thermoelectric trapping and assembly of six 2 μm PS beads using a 1.2 μm Si particle as a shuttle. **e** A schematic (left) and optical images (right) show the opto-thermoelectric trapping, transport and translation of a single 200 nm fluorescence PS bead from one 1.2 μm Si shuttle to another 1.2 μm Si shuttle. **f** A schematic (left) and optical images (right) show the opto-thermoelectric trapping and out-of-plane transport of a single 200 nm fluorescence PS bead using a single 1.2 μm Si particle as the shuttle. The dashed ellipses show the reference particles or assembly on the glass substrate. The grey arrows in **e** show the trajectory of the particles. The intersection of the green lines indicates the location of the laser beam. The trapping laser beam with a wavelength of 532 nm, an optical power of 60 μW, and a diameter of 2 μm at the focal plane was launched from the bottom. Scale bar: 5 μm **d**–**f**

Beyond SOTET of light-absorbing particles, where the heat generated from the particle can trap itself at the laser spot, we further show that this temperature field at the light-absorbing particle can be utilized to trap and manipulate non-absorbing particles. In this regard, the light-absorbing particle acts as an opto-thermoelectric shuttle, significantly expanding the applicability of SOTET to all sorts of synthetic or biological particles. Figure 3a displays the schematic of the thermoelectric field arising from the temperature gradient and ionic distribution around a heated Si particle. The temperature gradient perpendicular to the particle surface always points towards the particle centre, which generates a thermoelectric field $E^ \bot$ to trap other particles on the Si particle surface. Moreover, the temperature gradient parallel to the Si particle surface creates another thermoelectric field $E^\parallel$, which delivers the trapped particles towards the illuminated pole of the Si particle. The simulated thermoelectric field around the Si particle is shown in Fig. 3b. It should be noted that in addition to the trapping force arising from the thermoelectric field, an electrostatic repulsive force *F*_e_ from the positive Si particle surface and the weak optical scattering force *F*_scat_ are also considered. Considering the simple optical setup (NA: 0.5) and the low optical power (60 μW) used herein, we can ignore the optical gradient force. A force balance is achieved for the non-absorbing particle at the locations around the illuminated pole of the Si particle, and dynamic manipulation can be further achieved by controlling the Si shuttle.

To verify this shuttle concept, we used a single 1.2 μm Si particle as the opto-thermoelectric shuttle to manipulate non-absorbing polystyrene (PS) beads in a versatile manner. As shown in Fig. 3d, the thermoelectric field built by the Si shuttle drove the surrounding PS beads to migrate towards the Si surface and trapped them there. The Si shuttle can be driven dynamically to trap multiple PS beads. As an example, we drove the Si shuttle to assemble six PS beads into a 3D structure—five PS beads in plane and one PS bead out of plane (Fig. 3d; see also Video S7). We further demonstrate that the Si shuttle has the capability to manipulate nano-sized PS beads. As shown in Fig. 3e, a single 200 nm fluorescent PS bead was trapped and transported by a Si shuttle, and the PS bead was further delivered to another Si shuttle (see also Video S8). Moreover, the PS bead can be loaded onto the Si shuttle and transported in 3D (Fig. 3f).

### Long-distance opto-thermoelectric trapping and pulling

**Fig. 4:**
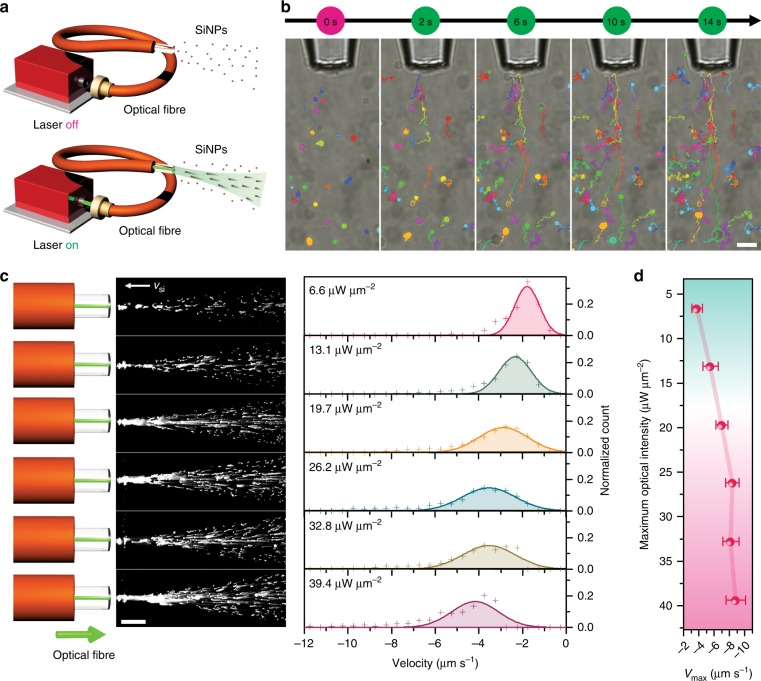
Long-distance opto-thermoelectric pulling and trapping. **a** Schematic showing the experimental setup for OTEP based on a laser-coupled optical fibre. **b** Long-distance opto-thermoelectric trapping of 500 nm Si particles using a tapered SM optical fibre. **c** OTEP trajectories (left) and probability distributions of the pulling velocities (right) of 800 nm Si particles in a 1 mM CTAC solution at optical intensities ranging from 6.6 to 39.4 μW μm^−2^. Individual frames from the recorded one-minute real-time videos were used to create the long-exposure images in the left panel. **d** The maximum OTEP velocity of 800 nm Si particles as a function of the maximum optical intensity. The 800 nm Si particles are pulled by an MM optical fibre with a diameter of 50 μm and an NA of 0.1 in **c**, **d**. The CTAC concentration was 1 mM. The optical intensities were calculated at the fibre tips. Scale bars: 5 μm **b**; 200 μm **c**

As discussed above, the optical trapping technique based on the OTEP effect is achieved through an objective lens to focus a laser beam that creates localized heating, where trapping takes place around the focus spot. We further demonstrate that, through a nearly collimated laser beam, we can overcome the limited working distance in traditional optical trapping technologies and thus achieve long-range trapping. As shown in Fig. 4a, an optical fibre with a small NA (0.1) is coupled to a laser source, with the fibre tip pointing towards the Si particle suspensions. When the laser is on, the Si particles are heated by the laser beam, pulled against the light path, and finally trapped at the fibre tip. As shown in Fig. 4b, a laser-coupled single-mode (SM) tapered optical fibre was used to demonstrate long-distance OTEP and trapping. We can see that when the laser was turned on, the 500 nm Si particles (rendered as coloured discs) within the illuminated regions became active, i.e., they moved towards the laser source and finally became trapped at the fibre tip (see also Video S9). However, the Si particles outside the illuminated regions (i.e., outside the light paths) underwent Brownian motion without any pulling phenomenon.

Moreover, we demonstrate that the OTEP force can be employed to optically pump a large number of particles up to a macroscopic distance. For the demonstration, a multi-mode (MM) optical fibre (core diameter: 50 μm, NA: 0.1) is coupled to a laser as the heating source. It should be noted that the OTEP velocity is a function of the CTAC concentration (see Fig. S7 and Video S10), which is consistent with our theoretical model on the thermoelectric field. Choosing an optimized CTAC concentration of 1 mM to maximize the pulling effect, we investigate the OTEP velocity of Si particles at different optical intensities. As shown in Fig. 4c, an increase in optical intensity from 6.57 to 39.42 μW μm^−2^ can significantly improve the temperature gradient on the Si particle surface during optical heating, and thus, the pulling velocity is remarkably increased (see also Video S11). It is noted that OTEP of Si particles at a macroscopic distance is achievable as long as the particles can be illuminated by the laser beam. In our experiment using a ×10 objective, we can observe a strong pulling effect on Si particles more than 1 mm away from the fibre tips. The slightly diverged beam (caused by the small NA) reduces the optical intensity when the fibre-particle distance increases, and thus, the pulling force on the Si particles is also reduced. We expect that the working distance can be significantly increased using a collimated laser beam, where the optical intensity becomes more uniform at different distances. It should be noted that, different from the trapping observed using sharp fibre tips^34–36^, the optical gradient force is ignorable at such low NA and low optical intensity in our work. The OTEP can be verified by our control experiment, showing that particle pulling cannot be achieved when CTAC surfactant is absent in the solution. Moreover, the CTAC concentration-dependent pulling velocity further suggests that the opto-thermoelectric field is dominant in the observed long-distance pulling.

In addition to the incident optical intensity, the observed pulling velocity of individual particles is also determined by their in-plane and out-of-plane locations, where the actual optical power illuminated on the particles and the hydrodynamic boundary effect should be considered, respectively. Thus, the statistics of the pulling velocities at the different optical intensities give a broad probability distribution at each optical power (right panel in Fig. 4c). To better reveal the optical-intensity dependency, we calculated the average pulling velocity of the 10% Si particles with the maximum pulling velocity, as shown in Fig. 4d. We can see that a maximum pulling velocity of 9 μm s^−1^ can be achieved at an optical intensity of 39.42 μW μm^−2^. It should be noted that at a significantly higher optical power, the Si particles can be pushed along the light path with a reversed migration direction, indicating that controllable optical pumping can be achieved by simply tuning the optical power.

## Discussion

We propose that the heat generated from a light-absorbing particle exerts a back action on the particle, which dominates the optical force and thus enables pulling the particle against the direction of light propagation. The underlying working mechanism of the pulling effect arises from the self-induced opto-thermoelectric field, which is determined by the surface temperature gradient instead of the incident photon momentum. By engineering the OTEP force via a localized heating spot, we manage to control the self-restoring behaviour of the light-absorbing particle to carry out 3D manipulation with simple and low-power optics. Moreover, the opto-thermoelectric field can be harnessed to manipulate non-absorbing particles based on the shuttle concept for expanded manipulation applications. Finally, we demonstrate that the OTEP effect can overcome the short working distance in traditional optical trapping. Our demonstrations with low-NA optical fibres reveal that the particles can be delivered over a macroscopic distance for trapping or pumping applications. In contrast to optical manipulation technologies based on momentum conversion, energy-oriented light-to-heat conversion is an entropically favourable physical process, with high opto-thermomechanical coupling efficiency achievable in many systems without rigorous optical engineering. Beyond low-power 3D optical trapping with simple optics, the optically controlled pulling and pushing effect over the macroscopic distance paves the way towards freeform 3D optofluidic and lab-on-a-chip systems without the need for physical channels.

## Materials and methods

### Synthesis of Si particles

The synthesis protocols of the hydrogen-terminated amorphous Si particles were previously reported^37^. Trisilane and *n*-hexane were added to a 10 mL titanium reactor placed inside a globe box filled with nitrogen. The amount of trisilane determines the size of the Si particles. A certain amount of *n*-hexane was used to maintain a reaction pressure of 34.5 MPa (5000 psi). After being sealed, the reactor was removed from the glove box and heated to a target temperature for 10 min for complete decomposition of the trisilane. After the reaction, the reactor was cooled to room temperature in an ice bath. The Si particles were then washed with chloroform by centrifuging at 8000 rpm for 5 min. The precipitate was collected and dispersed in chloroform before use. The hydrogen concentration inside the Si particles was tuned by controlling the reaction temperature. All the Si particles used in this work have a hydrogen concentration of ~5 at.%.

### Optical setup for SOTET

A 532 nm diode-pumped solid-state laser was used for optical heating. The laser was expanded with a ×5 beam expander and directed to an inverted microscope (Nikon Ti-E). After going through a ×100 oil-immersed objective (NA: 0.5–1.3), the laser beam was focused on a cover slip, where a chamber containing Si particles and CTAC solution was placed and sandwiched by a top coverslip. It should be noted that an NA of 0.5 was used in this experiment to reduce the optical gradient force. A while light was launched from the top for bright-field imaging.

### Optical setup for OTEP

A 532 nm diode-pumped solid-state laser was coupled into an optical fibre (tapered SM or MM fibre). The fibre was held horizontally by a home-made motorized stage and placed on the top of a coverslip. A drop of CTAC solution with Si particle suspensions was added on top of the coverslip. In Fig. 4c, the white light was turned off, and the laser beam shining on the Si particles generated a scattering signal for direct imaging.

### Particle trajectory analysis

The particle trajectories were evaluated from Blair and Dufresne’s open-source MATLAB adaptation of Crocker and Grier’s algorithms to study the colloidal particles^38^. The frames obtained from each video were converted into binary images after establishing thresholds on the intensity and size of each particle. A threshold on intensity removes the noise from images, and a threshold on the particle size removes the effect of particles a distance away from the focal plane (which appear larger than others) on the final data. Using Blair’s MATLAB functions, the position of each white blob (particle in the image) was retrieved, and an initial estimation of the position of each blob was found. This position was then improved by evaluating the centroid of each blob. The positions of particles in each frame, along with a timestamp, were concatenated vertically to obtain the tracks of particles over several frames, i.e., the position of each particle in the image as a function of time, thus resulting in the velocity of each particle. To reduce the dominance of Brownian motion on the velocity calculations, the trajectory of a particle was tracked over 15 frames, and the initial and final positions of the trajectory were used to evaluate the average velocity of the particle. The particle velocities thus tracked over the duration of the video in sets of 15 frames were used to generate a spatial average of the velocity as a function of the axial distance from the fibre. However, several constraints were imposed on the retrieved data. Based on our evaluation of the particle velocities as a function of the axial distance, the particles a distance away from the beam axis decreased the average velocity, as the Brownian motion was more dominant over the pulling force due to the decreased laser intensity. Therefore, only particles within a radial distance of 50 pixels from the fibre centre were considered for velocity evaluation. The concentration considered in the experiments is ideal to visualize the particle motion without any interference from the surrounding particles, which limits the amount of velocity data obtained from the videos to determine the exact spatial variation in velocity. To circumvent this problem, the axial distance was discretized into several intervals, and the velocities measured (at any time during the experiment) at any point within a given interval were averaged to obtain the average velocity as a function of the distance from the fibre tip. A Gaussian curve was fitted to determine the trend with respect to the laser power and surfactant concentration. A dual Gaussian curve was fitted when a reversal of migration of the particles was also observed.

### Trapping stiffness

The trapping stiffness of the Si particles was characterized by tracking the Brownian motion of the particles in SOTET. A monochromic charge-coupled device (CCD) camera (Andor) was used for particle tracking, with an exposure time of 10 ms and a duration of 30 s. The position probability of the particles was fitted by the Gaussian function to obtain the variance of the Brownian motion *σ*, and the trapping stiffness was calculated by *k*_T_ = 2*k*_B_*T*/*σ*^2^.

### Numerical simulation

A finite-element solver (COMSOL Multiphysics 5.2) was used to simulate the temperature profile of the Si particle. In the simulation, the incident Gaussian beam (NA: 0.5) was launched and used to irradiate a Si particle. The physics model involves electromagnetic waves and heat transfer in solids and fluids. Multiphysics coupling includes an electromagnetic heat source, boundary electromagnetic heat source, and temperature coupling. The boundary conditions for the electromagnetic wave and heat transfer were set as the scattering boundary and room temperature, respectively. The refractive index of amorphous silicon was taken from the literature^39^. The background medium was water with a refractive index of 1.33.

## Supplementary information

Supplementary Informantion

Video S1

Video S2

Video S3

Video S4

Video S5

Video S6

Video S7

Video S8

Video S9

Video S10

Video S11

## Data Availability

The data that support the plots within this paper and other findings of this study are available from the corresponding author upon reasonable request.
