# Supplementary material for: Opto-thermoelectric pulling of light-absorbing particles
Source: Light Sci Appl. 2020 Mar 6;9:34. doi: 10.1038/s41377-020-0271-6 (PMC7058623; doi:10.1038/s41377-020-0271-6)
Supplement: Supplementary file 1 — Supplementary Informantion [file 41377_2020_271_MOESM1_ESM.docx]

**Supplementary Information**

**Opto-thermoelectric pulling of light-absorbing particles**

Linhan Lin^1,2,3,*^, Pavana Siddhartha Kollipara^1^, Abhay Kotnala^1,2^, Taizhi Jiang^4^, Yaoran Liu^2,5^, Xiaolei Peng^2^, Brian A. Korgel^2,4^, and Yuebing Zheng^1,2,*^

^1^Walker Department of Mechanical Engineering, The University of Texas at Austin, Austin, TX 78712, USA

^2^Materials Science & Engineering Program and Texas Materials Institute, The University of Texas at Austin, Austin, TX 78712, USA

^3^State Key Laboratory of Precision Measurement Technology and Instruments, Department of Precision Instrument, Tsinghua University, Beijing 100084, People’s Republic of China

^4^McKetta Department of Chemical Engineering, The University of Texas at Austin, Austin, TX 78712, USA

^5^Department of Electrical and Computer Engineering, The University of Texas at Austin, Austin, TX 78712, USA

^*^E-mail: linlh2019@mail.tsinghua.edu.cn, [zheng@austin.utexas.edu](mailto:zheng@austin.utexas.edu)

**Supplementary Videos**

**Video S1: SOTET of single 500 nm Si particles in CTAC solutions with different concentrations.**

**Video S2: SOTET and in-plane transport of a single 1.2 μm Si particle on the glass substrate.**

**Video S3: SOTET and out-of-plane transport of a single 500 nm Si particle in 3D (2×).**

**Video S4: SOTET and in-plane transport of a single 500 nm Si particle in 3D (2×).**

**Video S5: SOTET and long-distance out-of-plane transport of a single 700 nm Si particle in 3D using a 40× objective (2×).**

**Video S6: SOTET and in-plane transport of a single 700 nm Si particle in 3D crossing a particle assembly on the substrate.**

**Video S7: Opto-thermoelectric trapping and assembly of six 2 μm PS beads using a 1.2 μm Si particle as a shuttle (2×).**

**Video S8: Real-time opto-thermoelectric trapping and transport of a single 200 nm fluorescence PS bead using a single 1.2 μm Si particle as a shuttle.**

**Video S9: Long-distance opto-thermoelectric trapping of 500 nm Si particles using a tapered single-mode optical fibre (2×).**

**Video S10: Long-distance OTEP of 800 nm Si particles using a multi-mode optical fibre at different CTAC concentrations (4×).**

**Video S11: Long-distance OTEP of 800 nm Si particles using a multi-mode optical fibre at different optical powers (4×).**

**Supplementary Figures**

**
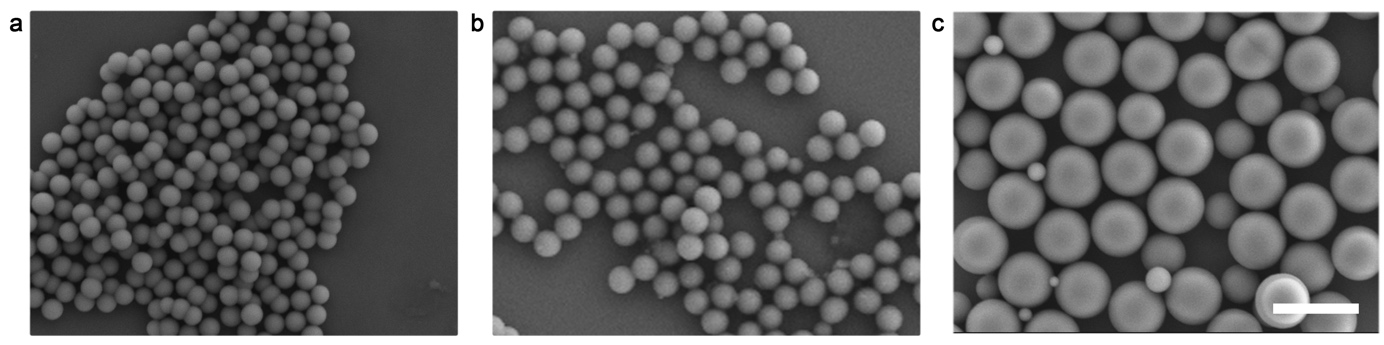
**

**Fig. S1. Scanning electron microscopic images of the Si particles.** Diameters: **a,** 500 nm; **b,** 700 nm; **c,** 1200 nm. Scale bars: 2 μm.


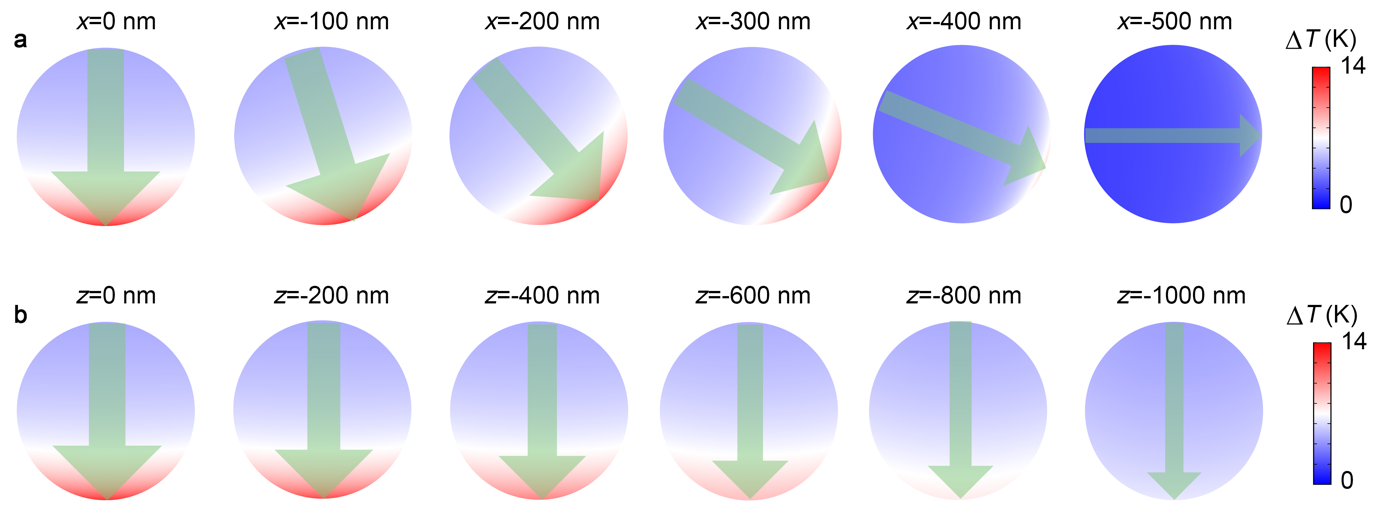


**Fig. S2. The dependency of surface temperature distribution as a function of particle location in relation to the incident laser beam. a,** Surface temperature increment $\Delta T$ at different *x*-offset between the particle center and the beam axis. **b,** Surface temperature increment $\Delta T$ at different *z*-offset between the particle center and the focus plane of the laser beam. The orientation of the green arrows gives the direction of the thermoelectric field, while the width of the green arrows represents the intensity of the thermoelectric field. The diameter of the Si particle is 700 nm, and the incident laser has an optical power of 60 μW.


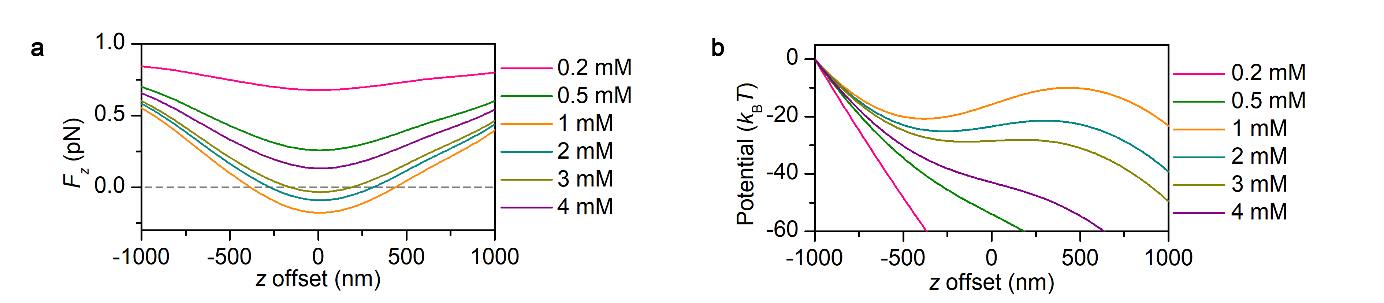


**Fig. S3. Out-of-plane force and trapping potential analysis.** **a,** The out-of-plane trapping force on a single 700 nm Si particle as a function of the CTAC concentration; **b,** The out-of-plane trapping potential on a single 700 nm Si particle as a function of the CTAC concentration.

**
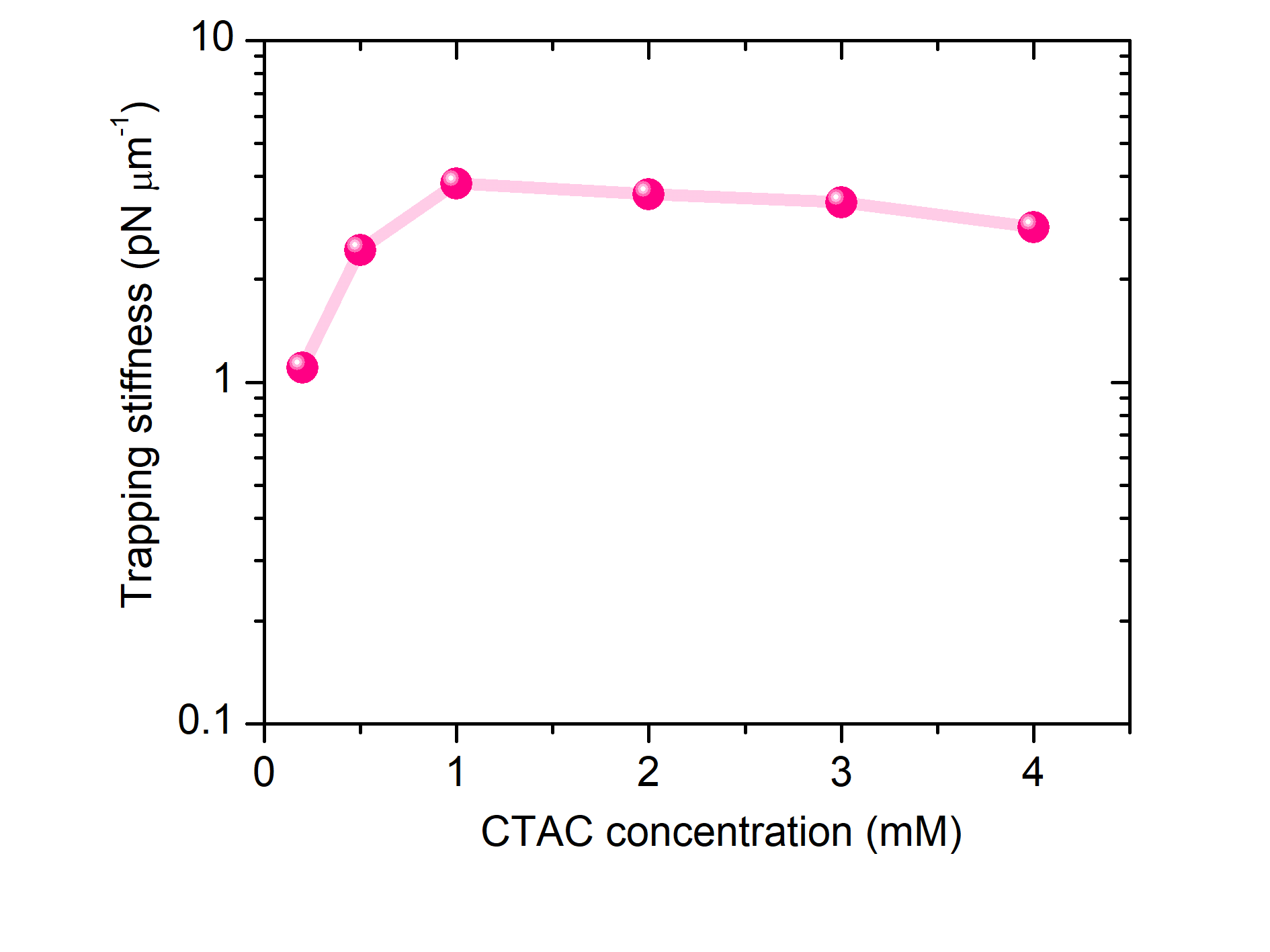
**

**Fig. S4. Theoretical trapping stiffness on single 700 nm Si particles as a function of CTAC concentration.**


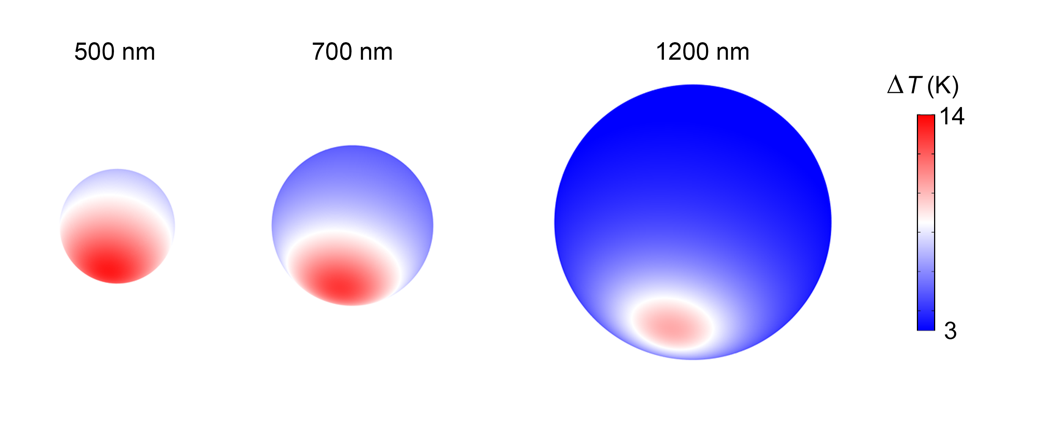


**Fig. S5. The surface temperature increment on the Si particle surface as a function of particle size.**


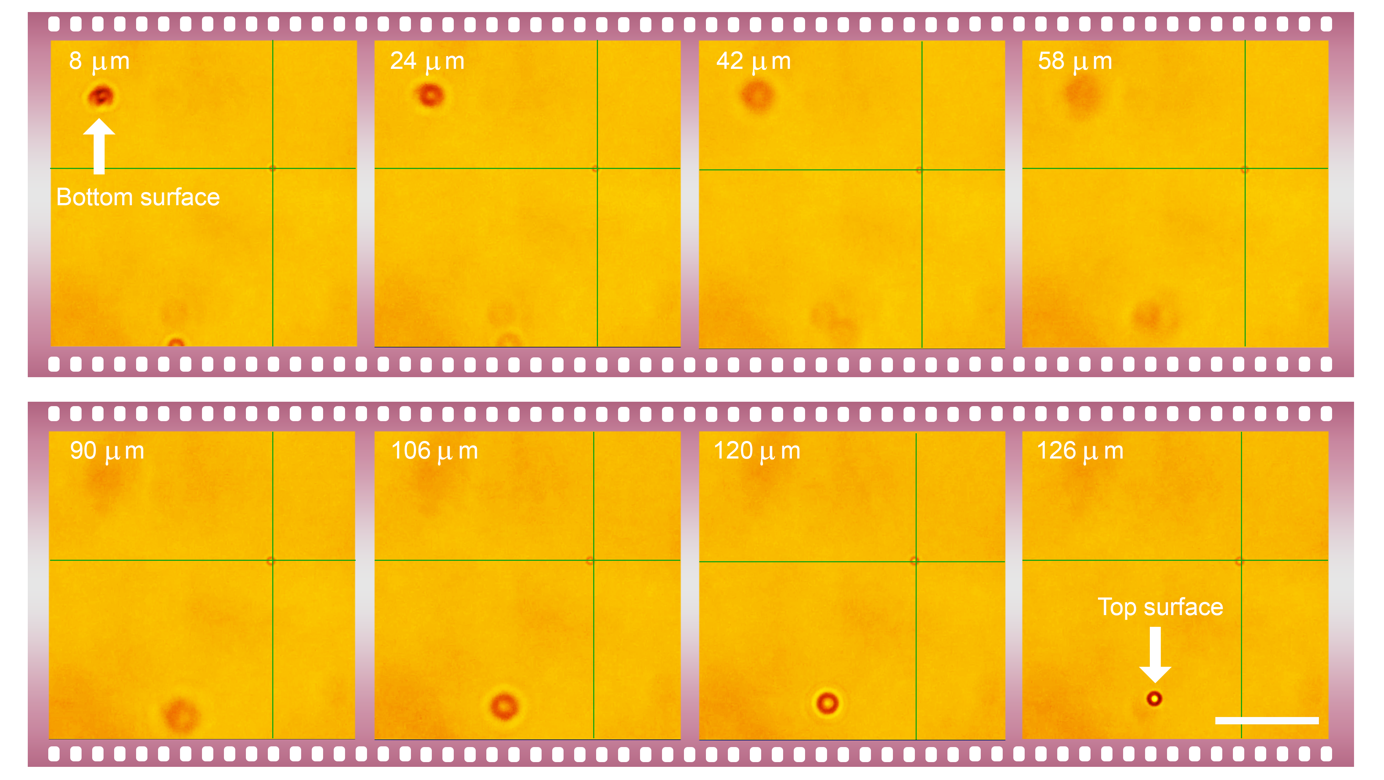


**Fig. S6. Out-of-plane transport of a single 700 nm Si particle using a 40 × objective (NA: 0.75).** Scale bar: 50 μm.


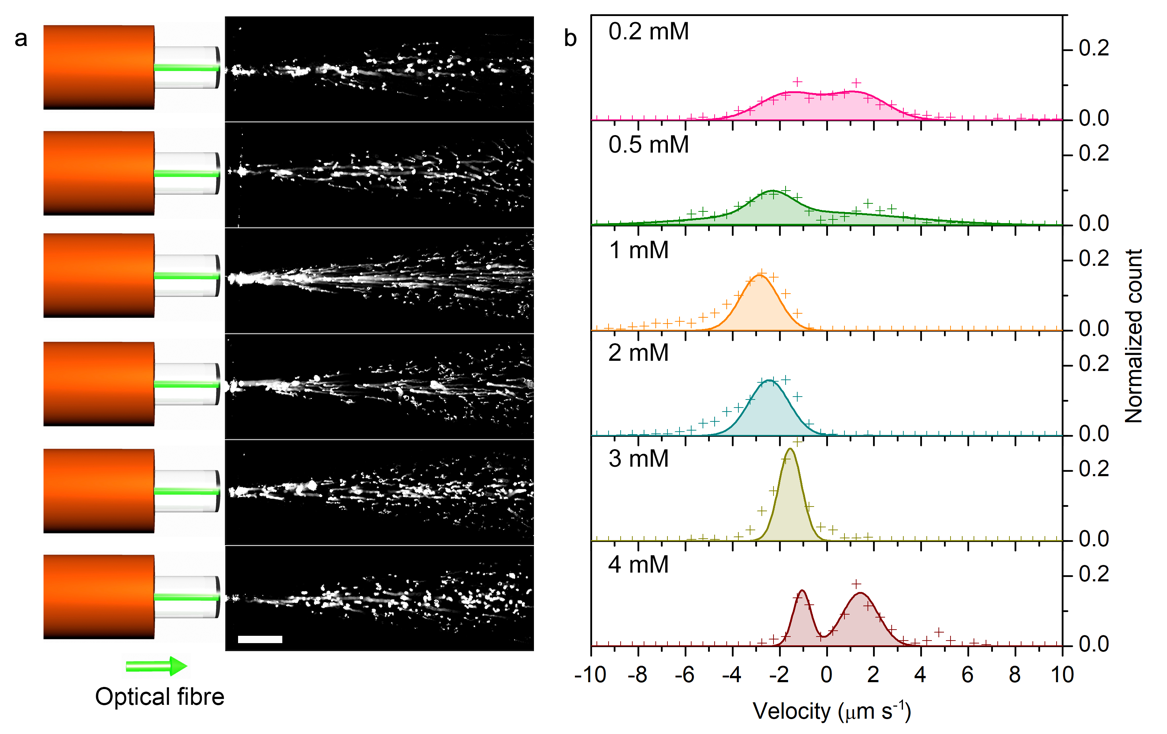


**Fig. S7. OTEP velocity at different CTAC concentrations.** **a,** OTEP trajectories of 800 nm Si particles in the CTAC solution with different concentrations. **b,** Probability distribution of the pulling velocity of 800 nm Si particles in the CTAC solution with different concentrations. The 800 nm Si particles are pulled by a multi-mode optical fiber with a diameter of 50 μm and an NA of 0.1. The maximum optical intensity at the fiber tip is 19.71 μW μm^-2^.

**Note S1**

Calculation of the opto-thermoelectric force and trapping potential

Opto-thermoelectric force on a silicon particle is due to the differential thermophoresis of the ions of surfactant (CTAC). The positive ions form micelles beyond a critical micellar concentration and act as macro ions. The resultant thermoelectric field around the particle can be simplified by assuming that the effect of individual surfactant ions on thermoelectric field is negligible. The resultant thermoelectric field, assuming that it stems only from macro-ions and counterions in the solution, is given as^1,2^

|  | $\boldsymbol{E}_{\mathbf{T}}=\frac{k_{B}T\boldsymbol{\nabla}\boldsymbol{T}}{e}\frac{\left[ S_{T_{2}}+\frac{Z_{1}S_{T_{1}}}{N_{\mathrm{agg}}} \right]}{1-\frac{Z_{1}S_{T_{1}}T}{N_{\mathrm{agg}}}}$ | (1) |
| --- | --- | --- |

where $\boldsymbol{E}_{\mathbf{T}}$ is the thermoelectric field, $T$is the temperature, $e$ is the electron charge, $N_{\mathrm{agg}}$ is the aggregation number of the micelles, $S_{T_{1}}, S_{T_{2}}$are the Soret coefficients of the micelles and counterions, respectively and $Z_{1}$is the effective valency of the micelles.

Three-dimensional temperature profile around the sphere is computed using electromagnetic and heat transfer modules in a commercial finite element analysis software, COMSOL Multiphysics$®$ version 5.3a. The resultant temperature gradient and the thermoelectric field obtained using equation (1) at the surface of the particle is resolved along the tangential and normal to the particle surface. While the normal component of the thermoelectric field is neutralized by the charge of the particle, the tangential component of the particle surface does not vanish^3^. The tangential component is numerically integrated around the particle surface along with the surface charge density of the particle as

|  | $\boldsymbol{T}\boldsymbol{E}_{\mathbf{Force}}= \int_{A} \sigma\boldsymbol{E}_{\mathbf{T}\boldsymbol{,}\boldsymbol{\parallel}}dA= \sum_{\theta=0}^{\pi} \sum_{\phi=0}^{2\pi} \sigma\boldsymbol{E}_{\mathbf{T}\boldsymbol{,\parallel}}a^{2}\sin\left( \theta\right)d\theta d\phi$ | (2) |
| --- | --- | --- |

where $\theta$ and $\phi$ are polar and azimuthal angles subtended by the point on the surface with respect to the particle center, $\sigma$ is the surface charge density and is approximated as $\zeta\epsilon/a$, $\zeta$ is the zeta potential of the solution, $\epsilon$ is the permittivity of the solution and $a$ is the particle radius. Thermoelectric force is then evaluated for different radial positions of the particle in the focal plane and the in-plane thermoelectric potential is calculated using $U\left( r \right)=-\int TE_{\mathrm{Force}}dr$.

**References**

1 Reichl, M., Herzog, M., Götz, A. & Braun, D. Why Charged Molecules Move Across a Temperature Gradient: The Role of Electric Fields. *Phys. Rev. Lett.* **112**, 198101 (2014).

2 Kollipara, P. S., Lin, L. & Zheng, Y. Thermo-Electro-Mechanics at Individual Particles in Complex Colloidal Systems. *J. Phys. Chem. C* **123**, 21639 (2019).
